# Providing Patients with Locally Advanced Cervical Cancer Access to Brachytherapy: Experience from a Referral Network for Women Treated in Overseas France

**DOI:** 10.3390/cancers14122935

**Published:** 2022-06-14

**Authors:** Rita Bentahila, Elie Rassy, Samir Achkar, Florence Sacino, Stefanos Bougas, Alexis Vallard, Vincent Vinh-Hung, Johan Encaoua, Pierre Gustin, Sylvie Mengue, Patricia Pautier, Philippe Morice, Sébastien Gouy, Sophie Espenel, Eric Deutsch, Cyrus Chargari

**Affiliations:** 1 Radiation Oncology Department, Gustave Roussy Cancer Campus, 94805 Villejuif, France; gbentahila@hotmail.fr (R.B.); samir.achkar@gustaveroussy.fr (S.A.); sophie.espenel@gustaveroussy.fr (S.E.); eric.deutsch@gustaveroussy.fr (E.D.); 2 Medical Oncology Department, Gustave Roussy Cancer Campus, 94805 Villejuif, France; elie.rassy@gustaveroussy.fr (E.R.); patricia.pautier@gustaveroussy.fr (P.P.); 3 Radiotherapy Department, University Hospital of Guadeloupe, 97159 Pointe-à-Pitre, France; florence.sacino@chu-guadeloupe.fr; 4 Radiotherapy Department, University Hospital of Martinique, 97200 Fort-de-France, France; stefanos.bougas@chu-martinique.fr (S.B.); alexis.vallard@chu-guadeloupe.fr (A.V.); vincent.vinh-hung@chu-martinique.fr (V.V.-H.); 5 Radiotherapy Department, University Hospital of Reunion Island, 97744 Saint-Denis, France; johan.encaoua@chu-reunion.fr; 6 Radiotherapy Department, Hospital Polynésie Française, 98714 Papeete, France; pierre.gustin@cht.pf (P.G.); sylvie.mengue@cht.pf (S.M.); 7 Surgical Oncology Department, Gustave Roussy Cancer Campus, 94805 Villejuif, France; philippe.morice@gustaveroussy.fr (P.M.); sebastien.gouy@gustaveroussy.fr (S.G.)

**Keywords:** cervical cancer, brachytherapy, radiation oncology, chemoradiation

## Abstract

**Simple Summary:**

Access to image-guided adaptive brachytherapy (IGABT) is limited in many regions in the world, thus leading to treatment care disparities. The aim of our retrospective study was to report the experience of a referral network for women with locally advanced cervical cancer (LACC) between radiotherapy facilities in Overseas France and our institution. This series of 64 patients shows the feasibility of a referral process for women with LACC who require IGABT with estimated probabilities of LC, progression-free survival, and overall survival (OS) of 94.6% (95% CI: 88.9–100.0%), 72.7% (95% CI: 61.1–86.5%), and 82.5% (95% CI: 72.0–94.5%). However, the centralization of this advanced technique to expert centers requires a well-defined workflow and appropriate dimensioning of resources to minimize overall treatment time.

**Abstract:**

Image-guided adaptive brachytherapy (IGABT) is part of the standard of care for locally advanced cervical cancer (LACC). Access to IGABT is limited in many regions, thus leading to treatment care disparities. We report the experience of a referral network for women with LACC between radiotherapy facilities in Overseas France and Gustave Roussy. This is a retrospective review of patients with LACC referred to Gustave Roussy, for pulsed-dose-rate (PDR) image-guided adaptive BT after initial radiation therapy in the French overseas between 2014 and 2021. Sixty-four patients were eligible to receive IGABT. Overall treatment time (OTT) was 60.5 days (IQR: 51–68.5). The median follow-up time was 17 months. At two years, estimated probabilities of LC, progression-free survival, and overall survival (OS) were 94.6% (95% CI: 88.9–100.0%), 72.7% (95% CI: 61.1–86.5%), and 82.5% (95% CI: 72.0–94.5%). In multivariable analysis, a D90CTVHR < 85GyEQD2 and a CTVHR volume > 40 cm^3^ were significant for poorer PFS (*p* = 0.001 and *p* = 0.009, respectively) and poorer OS (*p* = 0.004 and *p* = 0.004). The centralization of this advanced technique to expert centers requires a well-defined workflow and appropriate dimensioning of resources to minimize OTT.

## 1. Introduction

Cervical cancer is the fourth most common cause of cancer death in women worldwide [1,2]. The standard of care for treatment of locally advanced cervical (LACC) relies on definitive platinum-based concurrent chemo-radiotherapy followed by image-guided adaptive uterovaginal brachytherapy (IGABT). This treatment sequence yields to long-term local control of around 90% across all stages at 5-year [3,4,5,6]. However, disparities in health care access contribute to treatment heterogeneity and have a major impact on patient outcomes, leading to a high number of patients being undertreated despite potentially curative treatments do exist [7,8]. The establishment of referral networks could be a possible strategy to reduce treatment care disparities and increase access to modern standards of care, including brachytherapy techniques such as 3D guided treatment planning and interstitial implants [7,8,9,10].

Overseas France consists of French-administered territories that are located outside Europe and include Martinique, Guadeloupe, French Polynesia, and Reunion Island, located 6787, 6698, 15,719 km, and 9220 km away from France mainland, respectively. In comparison with metropolitan France, the incidence of cervical cancer is higher in these regions such as Guadeloupe (8.7 per 100,000), Reunion island (10.8 for 100,000 women), and French Polynesia (15.6 for 100,000 women) [11,12,13]. Surgery and chemoradiotherapy facilities are available in health care facilities in Overseas France. However, access to brachytherapy is very limited, with only one single brachytherapy center initiated in the Reunion Island in 2016 [13].

Given the lack of local access to brachytherapy and the major impact of brachytherapy on patient outcomes, over the years there has been a referral network established between Overseas France and some comprehensive cancer centers in mainland France in order to provide an access to IGABT. We report our experience of this referral network between a comprehensive cancer center and radiotherapy facilities in Overseas France.

## 2. Materials and Methods

### 2.1. The Patients

We collected from the medical records of patients referred, between February 2014 and September 2021, from Overseas France to the brachytherapy department of Gustave Roussy for a histologically confirmed LACC. Patients referred for salvage treatment of a recurrence were not eligible for this study analysis. All patients had their therapeutic strategy decided in a multidisciplinary meeting in Overseas France. Patients were treated with initial chemoradiation, then they were referred to Gustave Roussy to receive IGABT. This non-interventional study was conducted in accordance with ethical standards and approved by the local ethic committee (reference 2021-72).

### 2.2. Treatment Delivered in the Overseas France

At diagnosis, patients had a detailed gynecological examination, a pelvic magnetic resonance imaging (MRI), and a Computed Tomography (CT) or 18-Fluorodeoxyglucose Positons Emission Tomography/Computed Tomography (18-FDG PET/CT). A laparoscopic para-aortic lymph node dissection was proposed in patients without para-aortic lymph node uptake to rule out infra-radiological para-aortic lymph node extension. External beam radiotherapy (EBRT) was delivered in Overseas France through intensity-modulated radiotherapy (IMRT). Patients with macroscopic lymph node extension received a simultaneous integrated boost (SIB) or sequential lymph node boost, taking into account the contribution of IGABT [14,15]. Concurrent weekly cisplatin 40 mg/m^2^ was recommended.

### 2.3. Referral Process

Brachytherapy programmation was initiated after medical records of patients were received (emails or facsimile) by the brachytherapy unit, taking into account the expected date of radiotherapy completion. Regular reminders were sent by emails to highlight the importance of sending medical records to schedule brachytherapy as soon as possible in order to avoid treatment delays and to have an MRI performed at 40–45 Gy to guide the implant procedure. Transfers of patients to Gustave Roussy and medical appointments were organized in relationship with secretariats and social assistants.

Patients are covered by the French health insurance system and therefore all cancer treatment are free, including sanitary evacuation if required to deliver appropriate care.

### 2.4. Brachytherapy Procedure

Patients received a pulse-dose rate (PDR) brachytherapy boost at our institute, based on a 3D computerized assisted treatment planning through the PDR Selectron seed projector (Nucletron, an Elekta Company, Stockholm, Sweden) or Varian afterloader (Varian Medical System, Palo Alto, CA, USA), and using the vaginal mold technique, or standard applicators if interstitial implantation was required, to treat distal parametrial involvement. The decision to perform only intracavitary or a combined intracavitary/interstitial technique was guided by the clinical examination in the operating room, taking into account imaging findings [16,17,18].

After implant, an MRI was performed with applicator in situ, and definition of target volumes and organs at risk (OARs) was based on the guidelines of GEC-ESTRO (Groupe Européen de Curiethérapie, European Society for Radiotherapy and Oncology) [19]. High-risk clinical target volume included the gross tumor volume at the time of brachytherapy, cervix, and all areas suspected of residual disease at clinical examination and/or MRI. Intermediate risk clinical target volume (CTV_IR_) included a margin of 5 to 10 mm anatomical margin around CTV_HR_, taking into account initial tumor extent. Treatment planning aimed at delivering at least 60 Gy to 90% (D90) of the CTVIR and 85 Gy to 90% (D90) of the CTV_HR_, taking into account the contribution of EBRT after converting doses into equivalent doses per 2-Gy fractions (EQD2) using the linear-quadratic model with an α/β = 3 Gy for normal tissues and an α/β = 10 Gy for tumor. Minimal doses of 75, 85, and 75 Gy to the most exposed 2cc (D2cc) of rectum, bladder, and sigmoid colon were the dose constraints, respectively [20]. If there was no hematological contraindication, in order to deliver a total of at least 5 cycles of chemotherapy (total dose: 200 mg/m^2^ cisplatin), an additional cycle of chemotherapy could be delivered at the time of IGABT. If cisplatin was contraindicated, weekly carboplatin Area Under Curve 2 was proposed. It was intended that overall treatment time was as low as possible, ideally less than 55–56 days [21,22,23].

### 2.5. Follow-Up

After brachytherapy, patients were readdressed to their referring radiation oncologist in Overseas France and an MRI of the pelvis and para-aortic area was systematically prescribed 6 weeks after IGABT. Follow-up was carried out in Overseas France. If follow-up data were lacking, referring radiation oncologists were contacted to update the medical records.

### 2.6. Statistical Analysis

Overall survival (OS) and progression-free survival (PFS) were defined from the date of histological diagnosis to the date of progression or death, respectively. Survival without local, regional, and metastatic disease were defined from the date of histological diagnosis to the date of local, pelvic lymph node, and distant metastatic (including para-aortic) relapse, respectively. Patients without any of these events were censored at the time of the last follow-up. Relapse was classified as local, pelvic nodal, or distant (including para-aortic nodal). Treatment-related side effects were reported according to the Common Terminology Criteria for Adverse Events (CTCAE) v4 scale for acute and late side effects (occurring ≥3 months following treatment completion). The continuous variables were presented, and quantitative variables as mean (standard deviation, SD) or median (interquartile range, IQR) and discrete variables were presented as frequency counts and percentages. Bivariate comparisons of continuous variables were performed using the Student’s *t*-test or the Mann–Whitney or Kruskal–Wallis test; bivariate comparisons of percentages were performed using the Chi^2^ test or the Fisher’s exact test, as appropriate. Prognostic factors were identified by univariable analysis using the log rank test. All tests were two-sided and a *p*-value less than 0.05 was considered statistically significant. Variables significant in univariate analysis were entered into a multivariate Cox proportions hazards model, and 95% CIs for the estimated hazard ratios (HRs) were calculated. Statistical analyses were performed using SPSS statistics 20.0 (Statistical Package for SocialScience) for windows (an IBM company software, Chicago, IL, USA).

## 3. Results

Among 129 patients referred from Overseas France to our institute for brachytherapy, 64 patients fulfilled inclusion criteria and were included in the study for analysis (the 65 remaining patients were referred for adjuvant brachytherapy after surgery of an endometrial cancer). Fifteen patients were from Reunion Island, 10 were from Martinique, 14 were from Guadeloupe, and 25 were from Polynesia Island. The median age at diagnosis was 54 (range: 23–82) years. Patient characteristics, tumors, and treatments are presented in Table 1.

The median time of the interval between the first symptoms and the diagnostic confirmation was 13 weeks (range: 3 days–3.4 years). The main histology was squamous cell carcinoma (SCC) at 77%. Most patients presented an advanced stage of their disease: 11 (17%) had stage IIIC2 disease and four (6.2%) had stage IVB according to the Federation Internationale de Gynécologie Obstétrique (FIGO) version 2018. Twenty-four patients (38%) did not have 18-FDG PET/CT at baseline. Laparoscopic surgical para-aortic staging was performed in 20 patients (all were pN0). Among the 40 patients having 18-FDG PET/CT performed, 15 (37%) had pelvic lymph node 18-FDG uptake (N1) and 14 (35%) had para-aortic lymph node uptake (N2) according to the TNM (Tumor, Node, Metastasis) classification version 2021.

The median time interval between histological diagnosis and the first EBRT fraction was 94.3 days (range 21–400 days). Three patients (5%) had neoadjuvant chemotherapy (carbo-paclitaxel) because of initial oligo-metastatic disease. Sixty-two patients (97%) received cisplatin-based chemotherapy concurrently with EBRT, with a median of 5 cycles (range 3–9 cycles). Two patients (3.1%) did not receive concurrent chemotherapy because of medical contra-indications. The pelvic EBRT dose was 45 Gy through fractions of 1.8 Gy in 59 patients and five had an EBRT boost to the cervix to a total dose of 50.4–59.4 Gy (median dose 57.5 Gy). Sixteen patients had para-aortic irradiation, 14 of whom had para-aortic nodal metastatic uptakes at 18-FDG PET/CT and two for prophylactic irradiation. In 25 (39%) patients, medical records were addressed to our center before patients had completed their first week of EBRT and in 39 (61%) patients, medical records were sent later in the course of EBRT.

The median time interval between the completion of chemoradiation and brachytherapy initiation was 19.0 days (range 2–140 days).

All patients had their brachytherapy procedure performed after completing their whole EBRT course. At time of brachytherapy, the median CTV_HR_ volume was 29.9 cm^3^. Eighteen patients (28%) had a CTV_HR_ volume > 40 cm^3^, including 11 patients with CTV_HR_ volume > 50 cm^3^. Fifteen patients (23%) received a combined intracavitary/interstitial (IC/IS) technique. Compared to patients treated with only intracavitary technique, those patients treated with a combined IC/IS techniques had larger CTV_HR_ volumes (47.0 cm^3^ versus 27.9 cm^3^, *p* < 0.01), less frequent disease confined to the cervix (6.2% versus 44%, *p* = 0.01), and more frequent stages ≥ IIB (100% versus 87%, *p* = 0.045). Among the patients treated in 2019–2021, 13/34 (38%) had a combined intracavitary/interstitial technique. Median overall treatment time (OTT) was 60 days (range 37–176 days). Median OTT was 53 days for patients having their medical records referred prior to completing their first week of EBRT, versus 65 days for those referred later in the course of their treatment (*p* = 0.04).

At time of brachytherapy, 55/64 (86%) received an additional cycle of concurrent chemotherapy. The median total number of concurrent chemotherapy cycles was 6 (1–7). The median D_90_ CTV_HR_ was 85.6 GyEQD2 (range: 66.47–107.45 GyEQD2). Median Total Reference Air Kerma was 1.69 cGy cm^2^ h^−1^ (range: 0.538–1.687 cGy cm^2^ h^−1^). Median D2cc rectum, sigmoid, and bladder were 61 GyEQD2 (range: 49–71 GyEQD2), 59 GyEQD2 (44–73 GyEQD2) and 71 GyEQD2 (64–86 GyEQD2), respectively. An additional cycle of cisplatin was delivered at time of brachytherapy in nine patients (14%). Three patients with para-aortic lymph node uptake had adjuvant chemotherapy, based on four cycles of carboplatin and paclitaxel.

**Figure 1:**
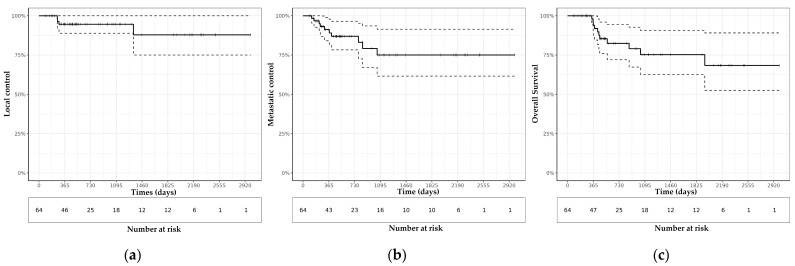
Kaplan-Meier survival curves for (**a**) Local control (LC), (**b**) Metastatic control (MC) and (**c**) Overall survival (OS) of 64 women with locally advanced cervical cancer (LACC) treated with image-guided adaptive brachytherapy (IGABT) after initial radiation therapy in the French overseas.

After a median follow-up of 17 months (range 2–98 months), 4 patients (6%) had isolated local relapse, and 3 had lymph node recurrence. The 2-year probability of local and regional control was 94.6% (95% CI: 88.9–100.0%) and 94.3% (95% CI: 86.7–100.0%). Ten patients had a distant metastatic failure, with or without regional or local disease, and the 2-year distant metastasis-free survival (MFS) was 86.9% (95% CI: 78.3–96.5%). Five (7.8%) patients were lost to follow-up after being readdressed to their treating radiation oncologist in Overseas France. The 2-year PFS and OS were 72.7% (95% CI: 61.1–86.5%) and 82.5% (95% CI: 72.0–94.5%), respectively (Figure 1).

Univariate models were fit to determine the OS, local failure, distant failure and PFS associated with histology, 18-FDG PET/CT use, FIGO stage, concurrent CT, CTV_HR_ volume, D_90_ CTV_HR_, and OTT. In univariate analysis, no impact of OTT or of time interval between EBRT and brachytherapy initiation was observed. Local control was poorer among patients with adenocarcinoma histology, with 2-year local control of 78.6% versus 100% for squamous cell carcinoma (*p* < 0.001) (See Table 2).

Variables known to affect survival were entered into a multivariate Cox proportions hazards model, and 95% CIs for the estimated hazard ratios (HRs) were calculated. In multivariable analysis, a D_90_CTV_HR_ < 85 Gy_EQD2_ was significant for poorer PFS (*p* = 0.001; HR: 7.4 (95% CI: 2.3–24.1) and poorer OS (*p* = 0.004, HR: 28.9; 95% CI: 2.9–279.7). A CTV_HR_ volume > 40 cm^3^ was significant for poorer PFS (*p* = 0.009; HR: 4.2; 95% CI: 1.4–12.5) and poorer OS (*p* = 0.004, HR: 13.9; 95% CI: 2.2–85.5). Adenocarcinoma histology was associated with poorer PFS (*p* = 0.013; HR: 4.0; 95% CI: 1.3–12.0).

Eleven patients (17%) had grade ≥2 acute gastrointestinal toxicity, 14 (22%) had acute genitourinary toxicity, and 13 (20%) had hematological grade ≥2 toxicity. Late toxicities were observed in 12 patients, mostly menorrhagia, pelvic pain, dyspareunia, and urinary incontinence. Five patients developed grade 3–4 toxicities, including one patient with vesicovaginal fistula and one patient with rectovaginal fistula. The patient with vesicovaginal fistula suffered with stage IIIC2 cervical cancer. She developed vesicovaginal fistula 4 months after treatment in the context of local recurrence of 4cm and was treated with a total hysterectomy with bilateral annexectomy. The other patient with initial T4a disease developed recto-vaginal fistula 14 months after treatment with metastatic recurrence. She was treated with anterior pelvectomy with Bricker derivation and colostomy.

## 4. Discussion

Brachytherapy increases the probability of cure in patients with LACC [24,25,26]. An analysis from the Surveillance, Epidemiology, and End Results (SEER) database demonstrated that brachytherapy treatment was independently associated with better cancer-specific survival (hazard ratio [HR], 0.64), and OS (HR 0.66) [27]. In addition, implementation of IGABT was associated with better disease control and lower complication probability, through dose escalation to the tumor in parallel with better sparing of organs at risk [20]. The therapeutic process is however complex and requires a health care structure with access to combined intracavitary/interstitial brachytherapy procedures. Adequate training of physicians, physicists and radiotherapy technicians is required, as published data showed a correlation between increased experience, ability to fulfill planning aims, and patient outcome [28]. The lack of radiotherapy-brachytherapy facilities is a major concern in many parts of the world. Various strategies can be applied to increase access to high-level treatments including multicenter cooperation [7].

A collaboration was progressively established between some radiotherapy centers in Overseas France and Gustave Roussy to perform IGABT on women with LACC. Indeed, centralization to expert centers for brachytherapy delivery is an appealing strategy. In this series, most patients received concurrent chemotherapy, in line with current guidelines based on large meta-analyses showing that concomitant chemotherapy improved OS and PFS [29]. In addition, the median D90 CTV_HR_ dose was >85 Gy_EQD2_, in relation to the very high local probability that is reported in this series, despite patients presenting, in most cases, at an advanced stage of their disease [30]. Despite delays, patient outcome remained high, with 2-year local control rate of 94% that can be attributed to the process of dose escalation and possibly the use of a combined IC/IS technique in 20% of patients [20]. The use of concurrent chemotherapy at time of brachytherapy in 86% of patients may also have contributed to these results [31]. Though local control was high, even among patients with the bulkiest residual tumors, IC/IS use was not sufficient to completely reverse the higher risk of relapse in this subgroup of patients who still had a high risk of distant relapse. We did not have enough patients in this cohort to examine in subgroup analysis the benefit of interstitial procedures according to tumor stage. Other authors have clearly shown, from larger cohorts, the benefit of a combined IC/IS to achieve high local control probability in advanced tumors [32]. Our data highlight the need for systemic intensification in these patients with bulky residual disease, taking into account the high risk for metastatic event. The development of novel radiosensitizers is also to be encouraged in the context of poorly responding tumors [33].

This series shows that a multicenter collaboration is possible, even in complex situations with large geographic distances and maximal jet lags (12 h between Papeete and Paris), to provide to patients a high-quality local treatment based on modern standards.

However, there are still substantial challenges to overcome to improve workflows. The median OTT was 60 days (range 37–176), which is longer than the usual recommendation of 50–56 days [25]. We found an impact of precocity of referral process (prior to or after the patients completed their first week of EBRT) and OTT. The median time interval between the completion of EBRT and brachytherapy initiation was 19.0 days (range: 2–140 days). Thirty-four patients (53%) were treated in 2019–2021 and OTT was impacted by COVID-19 crisis, in the context of deleterious impact on health care structures [34]. An optimal process of patient navigation between Overseas France and our institute requires organizing the transfers ahead and referring medical records from the early steps of treatment.

**Figure 2:**
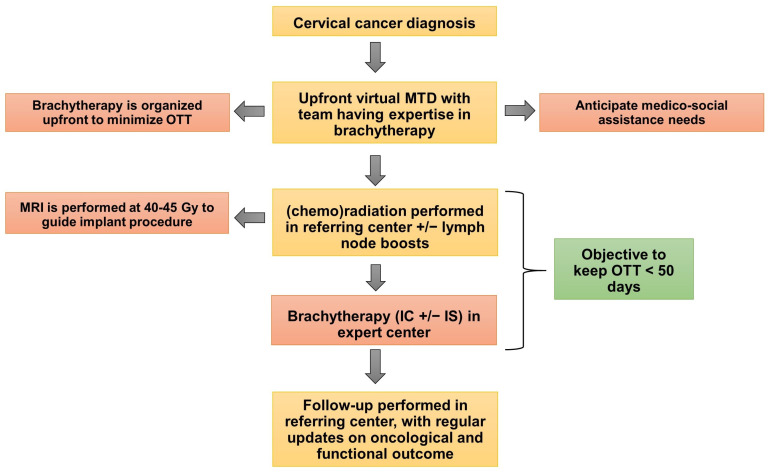
Example of a process of patient navigation for brachytherapy. Abbreviations: MRI: magnetic resonance imaging, IC: intracavitary, IS: interstitial, MTD: Multidisciplinary.

A well-defined workflow is necessary to minimize OTT, which is an important factor for local control, and this collaboration was at its beginning informal, without a defined referring procedure. A proposition for an optimal process of patient navigation for uterovaginal brachytherapy is shown in Figure 2. A long waiting list for brachytherapy treatment may contribute to OTT, suggesting that centralization of brachytherapy cases to expert centers requires adequate dimensioning of resources in terms of medical and paramedical staff, as well as easy access to an operating room, post-implant MRI, and treatment rooms (for PDR treatments) [35]. In addition, there are potential medico-social problems for patient navigation, and social assistance is often required and represents a key element of the overall patient care, as these patients need to stay in mainland France during their treatment. In parallel with the establishment of referral networks for complex combined intracavitary/interstitial procedures, it is also crucial to develop brachytherapy in Overseas France, as recently achieved in the Reunion Island, at least for cases that can be treated with intracavitary procedures.

Our study has some limitations. First, we only looked at patients referred to our center and therefore the proportion of patients receiving brachytherapy as part of their local treatment in Overseas France remains uncertain. Some patients may refuse brachytherapy because of the long travel distance, personal reasons, or financial problems. Second, our median follow-up was short, in line with the fact that most patients were treated in the past two years. In addition, this was not a prospective registration and therefore patients included in this series represented a partial but unselected subset of patients referred to our center. Finally, follow-up was performed by referring centers after brachytherapy and we did not systematically receive details of the follow-up consultations.

## 5. Conclusions

Our series shows the feasibility of a referral process for increasing access to modern concepts of image-guided brachytherapy. The impact of brachytherapy on patient outcomes should incite health care policymakers to increase access to brachytherapy and promote referral to expert centers applying modern concepts of IGABT and investing in advanced combined intracavitary/interstitial brachytherapy procedures [36]. There are, however, limitations to such referral procedures, and centralization requires that a well-defined workflow is available and appropriate dimensioning of resources to minimize OTT. In parallel with this process of referring complex cases to expert centers, it is important to develop expertise for brachytherapy in all parts of the world to increase access to this crucial component of patient treatment.

## Figures and Tables

**Table 1:** Patient, disease, and treatment characteristics.

Variable		Total (*n* = 64)
Age at diagnostic (years)	Median (min-max)	54.0 [23.0; 82.0]
Histology	SCC	49 (77%)
	Adenocarcinoma	14 (22%)
	Other	1 (1.6%)
Tumor (T)	T1b1	1 (1.6%)
	T1b2	5 (7.8%)
	T1b3	1 (1.6%)
	T2a	8 (12%)
	T2b	32 (50%)
	T3a	2 (3.1%)
	T3b	3 (4.7%)
	T4	12 (19%)
Nodal (N)	N0	22 (34%)
	N1	28 (43.7%)
	N2	14 (21.8%)
Metastasis (M)	M0	60 (94%)
	M1	4 (6.2%)
FIGO stage	IB2-IIA	6 (9.5%)
	IIB	14 (22%)
	IIIB-IV	43 (68%)
18-FDG PET/CT (%)	Negative	11 (17%)
	Pelvic node	15 (23%)
	Para-aortic	14 (22%)
	Not done	24 (38%)
Cervical external radiotherapy boost	Yes	5 (7.8%)
Volume CTV_HR_	Median (min-max)	29.9 [18.4; 44.4]
D90 CTV_HR_	Median (min-max)	85.6 [66.5; 107]
D90 CTV_IR_	Median (min-max)	67.4 [55.1; 78.6]
D2cc Rectum	Median (min-max)	61.8 [49.8; 80.3]
D2cc Sigmoid	Median (min-max)	59.6 [43.4; 73.4]

Abbreviations: CT chemotherapy; RT radiotherapy; 18-FDG PET/CT: 18-fluorodeoxyglucose positron emission tomography/computed tomography; CTV_HR_: high-risk clinical target volume; CTV_IR_: intermediate risk clinical target volume; D90: minimal dose to 90% of the volume; D2cc: dose to 2cc, FIGO: ‘International Federation of Gynecology and Obstetrics; GEC-ESTRO: European Brachytherapy-European Society for Radiation Oncology; SCC: squamous cell carcinoma; OTT: total treatment time.

**Table 2:** Univariate analyses of factors predicting for outcomes (Kaplan Meier estimates). Only factors reaching significance (*p* < 0.05) are reported.

	*n*	Overall Survival		Local Failure		Distant Failure		Progression-Free Survival	
		2Y (95% CI)	*p*-Value	2Y (95% CI)	*p*-Value	2Y (95% CI)	*p*-Value	2Y (95% CI)	*p*-Value
Histology Adenocarcinoma Other	14 50	70.0% (46.7; 100.0) 85.8% (74.9; 98.4)	0.12	78.6% (59.8; 100.0) 100.0% (100.0; 100.0)	<0.001	73.9% (51.9; 100.0) 90.7% (82.4; 99.9)	0.44	47.6% (26.7; 84.9) 81.4% (69.4; 95.4)	0.016
CTV_HR_ volume (cm^3^) ≤40 >40	46 18	94.4% (87.1; 100.0) 44.0% (20.9; 92.5)	<0.001	95.0% (88.5; 100.0) 93.8% (82.6; 100.0)	0.95	94.9% (88.3; 100.0) 64.4% (43.1; 96.3)	<0.01	87.5% (77.8; 98.4) 27.7% (9.39; 81.5)	<0.001
CTV_HR_ D90 dose <85 Gy ≥85 Gy	26 38	67.8% (50.5; 91.0) 96.2% (89.0; 100.0)	<0.001	96.0% (88.6; 100.0) 93.5% (85.3; 100.0)	0.75	78.8% (63.9; 97.3) 93.0% (84.0; 100.0)	0.043	55.5% (37.6; 81.9) 86.6% (75.2; 99.8)	<0.01

Abbreviations: 2 Y: 2 years, CI: confidence interval; CTVHR: high risk clinical target volume; D90: minimal dose to 90% of the volume.

## Data Availability

Research data are stored in an institutional repository and will be shared upon request to the corresponding author.
